# Broadly applicable methods for the detection of artefacts in electroencephalography acquired simultaneously with hemodynamic recordings

**DOI:** 10.1016/j.mex.2023.102376

**Published:** 2023-09-14

**Authors:** Rachel Nuttall, Aya El Mir, Cilia Jäger, Svenja Letz, Afra Wohlschläger, Gerhard Schneider

**Affiliations:** a Department of Anesthesiology and Intensive Care, School of Medicine, Technical University of Munich, Klinikum rechts der Isar, Munich 81675, Germany; b Department of Neuroradiology, School of Medicine, Technical University of Munich, Klinikum rechts der Isar, Munich 81675, Germany; c New York University Abu Dhabi, Engineering Division, Saadiyat Marina District, Abu Dhabi, United Arab Emirates

**Keywords:** Artifind, EEG-fMRI, Gradient, Pulse, Artefact, Cardioballistic, ECG, Ballistocardiac

## Abstract

Electroencephalography (EEG) data, acquired simultaneously with magnetic resonance imaging (MRI), must be corrected for artefacts related to MR gradient switches (GS) and the cardioballistic (CB) effect. Canonical approaches require additional signal acquisition for artefact detection (e.g., MR volume onsets, ECG), without which the EEG data would be rendered uncleanable from these artefacts.

• We present two broadly applicable methods for artefact detection based on peak detection combined with temporal constraints with respect to periodicity directly from the EEG data itself; no additional signals are required.

We validated the performance of our methods versus the two canonical approaches for detection of GS/CB artefact, respectively, on 26 healthy human EEG-functional MRI resting-state datasets. Utilising various performance metrics, we found our methods to perform as well as – and sometimes better than - the canonical standard approaches. With as little as one EEG channel recording, our methods can be applied to detect GS/CB artefacts in EEG data acquired simultaneously with MRI in the absence of MR volume onsets and/or an ECG recording. The detected artefact onsets can then be fed into the standard artefact correction software.

## Graphical abstract

**Figure:**
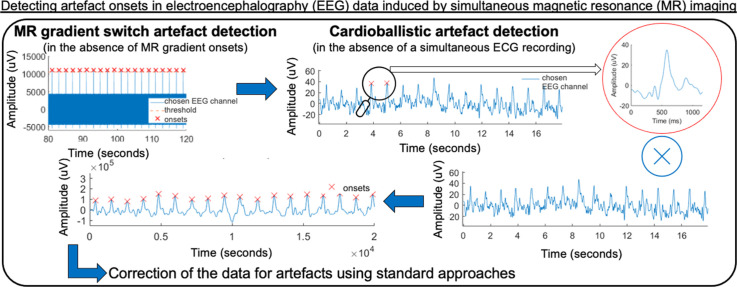

Specifications Table

**Table:** 

Subject area:	Neuroscience
More specific subject area:	Artefact removal
Name of your method:	Artifind
Name and reference of original method:	Sijbers, J. et al. (1999) ‘Restoration of MR-induced artifacts in simultaneously recorded MR/EEG data’, *Magnetic Resonance Imaging*, 17(9), pp. 1383–1391. Christov, I. I. (2004) ‘Real time electrocardiogram QRS detection using combined adaptive threshold’, *BioMedical Engineering Online*, 3(1), pp. 1–9.
Resource availability:	https://github.com/rachelnutt3012/Artifind

## Method details

### Background

The electroencephalogram (EEG) offers a non-invasive and direct measurement of neural activity in humans with a remarkable temporal resolution on the order of milliseconds [4,24]. Electrodes placed on the scalp of the participant measure the electrical activity generated by multiple underlying neural populations, each comprised of thousands of synchronously active pyramidal neurons perpendicularly aligned with the surface of the cortex [14,30]. As a result, the spatial resolution of EEG is poor, which is considered one of its major drawbacks.

Measurement of the Blood-Oxygenation-Level dependent (BOLD) signal via functional magnetic resonance imaging (fMRI) is also a frequently used tool for the non-invasive measurement of neural activity in humans, albeit indirect. This highly localised signal reflects magnetic field distortions, taking advantage of the local magnetic susceptibility effects of deoxygenated haemoglobin [5]. The BOLD signal has been closely tied to fluctuations in local field potentials generated by neural activity [[16], [17], [18], [19], [20],^22^,^26^,31], and is frequently used as a proxy of neural activity, with the advantage of a very high spatial resolution. On the downside, the BOLD signal possesses a relatively poor temporal resolution on the order of seconds [21], depending on the exact sequence and parameters in use.

Clearly, both EEG and fMRI possess advantages and disadvantages when it comes to the measurement of neural activity in humans. Over the last decades, technological advances have enabled the simultaneous acquisition of both signals i.e., simultaneous EEG-fMRI [2,^3^,^10^,^11^,^13^,^15^,^25^,29]. This approach has been proposed to offer a ‘best of both worlds’ scenario – the temporal specificity of EEG with the spatial specificity of fMRI in one simultaneous dataset [23,^27^,28].

However, when acquired simultaneously with magnetic resonance imaging, EEG data is fraught with extreme artefacts induced by the MR environment. The most extreme artefact is the gradient switch artefact, consisting of a sum of sinusoids of varying phase delays, amplitudes and frequencies [8] originating from the time-varying fields as created by the radio-frequency pulse for spin excitation and the switching of magnetic field gradients for slice selection. The second artefact is the cardioballistic (CB) artefact, caused by movements of electrodes due to head motion and scalp pulsations related to cardiac activity [2]. Both artefacts must be detected and removed from the data before further analysis.

## Canonical approaches to artefact detection

### GS artefact detection

The most frequently used approach to GS artefact detection is to record the acquisition onsets of each volume/slice, providing a marker of gradient switch onsets [32]. Henceforth, we will refer to this method as the ‘canonical’ approach to GS artefact detection.

### CB artefact detection

The most frequently used approach to CB artefact detection is to record a simultaneous ECG, from which the R-peaks of each QRS cycle is marked and used as a marker of cardiac beat onsets [6]. Henceforth, we will refer to this method as the ‘canonical’ approach to CB artefact detection.

### Rationale for our development of alternative approaches to artefact detection

Unfortunately, however, the complexity of the setup for a simultaneous EEG-fMRI experiment means that sometimes these signals required by canonical approaches for artefact detection aren't recorded or they are rendered unusable due to saturation as a result of inappropriate amplifier settings. Simultaneous EEG-fMRI is a time-consuming and costly data collection method, often meaning that EEG-fMRI studies have low sample sizes. Even one EEG-fMRI dataset acquired without the additional signals and without the ability to detect and clean the data for GS/CB artefacts would be a significant loss. We propose here a method that can detect these artefacts in datasets without these additional signals.

Two other methods have been previously proposed for GS and CB artefact onset detection in the absence of additional recordings but come with their own limitations. The GS artefact has been previously modelled as a linear time-invariant model consisting of three superimposed pulse response functions in the x, y, and z gradient directions [9]. However, from our observations in our data, the onsets of the time-invariant GS artefact can be easily identified from the raw EEG data and used directly and efficiently for the widely applied average artefact subtraction and residual correction methods, without any need for more complex modelling estimations. Some commercial systems also offer in-built software for the detection of GS artefact onsets, albeit not open source. Another approach [12] took advantage of the polarity difference between the EEG signal acquired at multiple left and right-sided frontotemporal electrodes and used this voltage difference to identify CB peaks. Although showing excellent performance at replicating peak detection as achieved with the canonical approach of ECG-based R-peak detection [6], this method requires bipolar recordings with most optimal performance on high-density EEG recordings. The method also showed good performance on EEG recordings with 64 channels, albeit reduced, with performance on 32 channel recordings unreported. Although 64 channel recordings are currently common practice in EEG-fMRI, unipolar datasets or datasets with a limited number of channel recordings without an ECG will require alternative methods for the detection of the CB artefact from the EEG.

We present here two methods for the detection of GS and CB artefacts, respectively, in EEG data acquired simultaneously with MRI. These methods allow one to detect these artefacts directly in the EEG data itself with as little as one EEG channel recording, without any additional signals such as MR volume triggers or an ECG recording. Hence, EEG datasets that cannot be cleaned for these artefacts using canonical approaches can be recovered using our methods.

### Our artefact detection methods

**Fig. 1:**
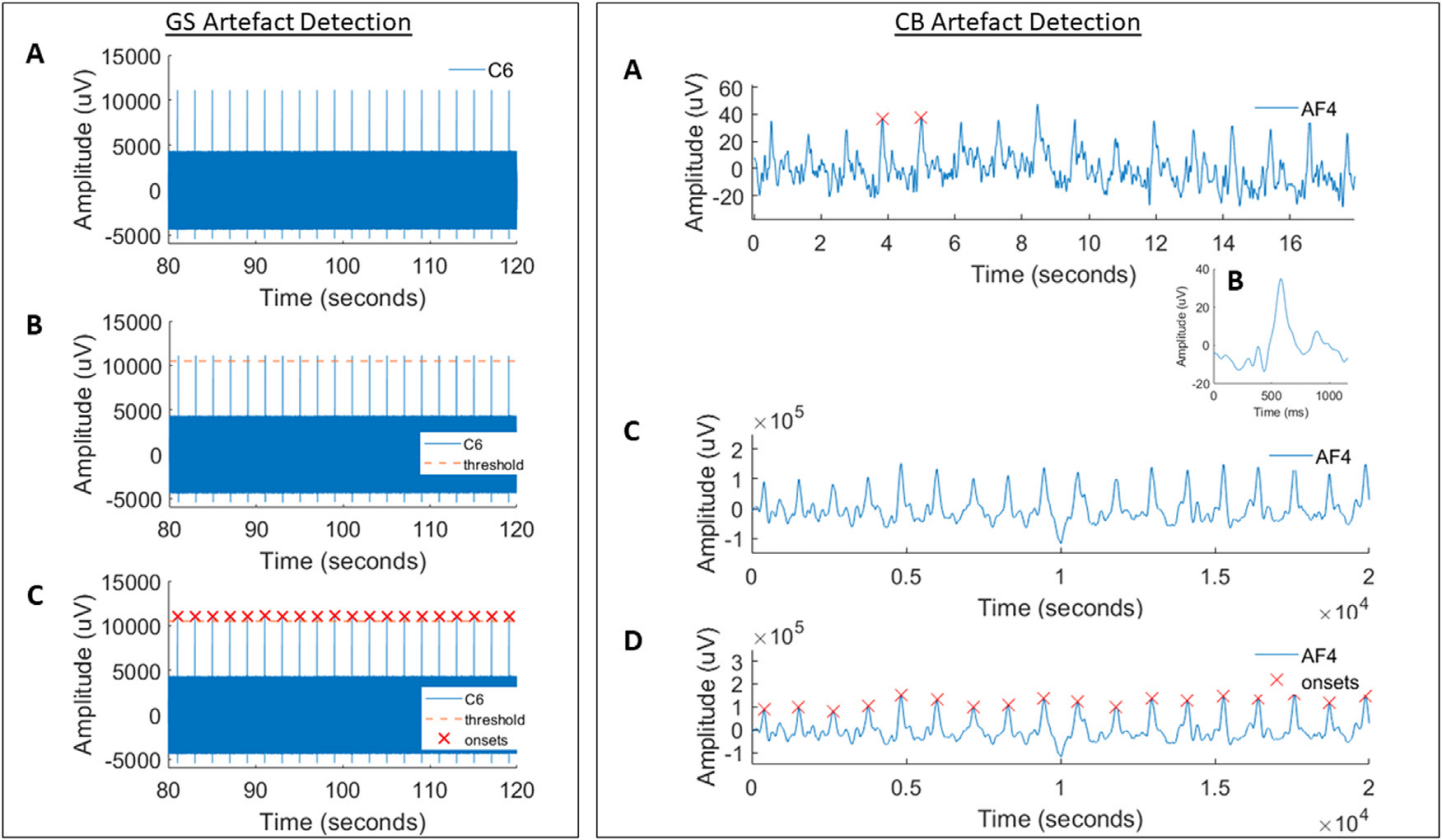
Our proposed methods for artefact detection. Left Panel: Gradient switch (GS) artefact detection. (A) An example segment of artefactual data from electrode C6 is shown. (B) An iterative threshold is applied, lowering the threshold on each iteration until a number of peaks equal to the expected number of artefacts can be automatically found and labelled as the artefact onsets (the red crosses as shown in (C)). These can then be entered into canonical software for artefact correction. Right Panel: CB artefact detection. (A) shows an example segment of artefactual data from electrode AF4. Two clearly discernible and consecutive peaks are first labelled. Using the information from these peaks, other peaks (i.e., artefact onsets) in the segment are automatically identified and a mean across all artefacts is taken (as shown in (B)). This mean artefact is convolved with the data segment (C) and peaks are automatically identified from the convolved signal (D). These are the artefact onsets that can then be inputted into canonical software for artefact correction.

See Fig. 1 for an overview illustration of our methods.

### Method 1: gradient switch (GS) artefact detection

Our first method detects the GS artefact onsets from the EEG data itself. Based on some basic user-input parameters (TR, number of volumes acquired, number of slices, number of dummy scans and the sequence used) the correct duration and number of artefacts that should be detected is calculated. In the case of volume-based GS artefact detection, the number of artefacts is equal to the number of volumes and the artefact duration is equal to the TR. In the case of slice-based GS artefact detection, the number of artefacts is equal to the number of volumes acquired multiplied by the number of slices acquired and the artefact duration is the TR divided by the number of slices, with no break before the next volume acquisition as occurs in some acquisition schemes (of course, these parameters are also slightly different for multiband sequences; for multiband sequences, you will need to instead enter the number of radiofrequency pulses used to sample all slices as your ‘number of slices’).

First, the median maximum amplitude value across all channels is calculated. This value is used as an initial threshold (Fig. 1, left panel, B). The software iterates through each EEG channel recording, labelling all peaks above this threshold (Fig. 1, left panel, C). Once the peaks have been labelled, the time between each peak is calculated, with the requirement that the time between the peaks is roughly equal to the expected artefact duration. If this requirement is not fulfilled at the current channel, the software skips to the next channel. ‘Roughly’ is defined as within two sampling points of the expected duration, giving flexibility for minor drifts in artefact peaks. If the most frequently observed value (mode) of distance between the peaks is roughly equal to the expected artefact duration and the number of peaks detected is roughly equal to the number of artefacts expected (+/−7 of the expected number of artefacts due to the presence of artefacts from dummy scans) then the user is shown the channel data with the labelled detected artefacts and asked if they wish to continue with artefact detection using this channel. Otherwise, the user can repeat the above steps on the next channel, and so on, until they find a channel with which they are happy to continue. As an example, some channels are affected more by initial spurious calibration procedures of the MR scanner, and these are sometimes (besides dummy scans) labelled as peaks. Users can allow the method to scroll through the channels until only the expected number of artefacts plus those induced by dummy scans are detected.

Once a channel has been chosen, a number of the first GS onsets are deleted, equal to the number of dummy scans. This procedure ends in a vector of onsets of detected GS artefacts. This vector of onsets can then be entered into standard correction algorithms.

### Method 2: cardioballistic (CB) artefact detection

Our second method detects the CB artefact directly at the scalp from the EEG recordings with no assumption as to the delay time. The data is initially high pass filtered to above 1 Hz (FIR filter, transition bandwidth 1, order of 5) to remove any slow fluctuations in the data. The filtered data is then segmented into non-overlapping segments of a length inputted by the user. The purpose of segmentation is to account for changes in the shape and amplitude of the artefact over time, as induced by motion. For our analysis, we used a fixed segment length of 20 s (considerations of the segment length can be found in section 5).

For each segment, a reference artefact is manually identified. The user is shown a plot of the data segment, and they must manually select the peak of two consecutive and clear CB artefact peaks (Fig. 1, right panel, A). The first channel is initially displayed, but the user can scroll through all available EEG channels until the channel that best presents the artefact for this segment is found (the chosen channel for this segment), and two peaks can be labelled. The distance (‘RtoR’) between the two labelled peaks is calculated, and the data surrounding the two artefacts are extracted (i.e., the peak of the artefact - RtoR/2 to the peak of the artefact + RtoR/2). The artefacts are then zero-meaned and the mean across the two is taken as the reference artefact.

To further increase the applicability of this reference artefact to all artefacts in this segment of data from the chosen channel, all peaks in the segment with a minimum peak distance equal to the RtoR minus 10 sampling points (to account for variability) are detected. A mean artefact is derived from all labelled artefacts with a reasonable correlation coefficient with the reference artefact (a Pearson's correlation coefficient *r* of at least 0.7).

This mean artefact (the convolution kernel; Fig. 1, right panel, B) is then convolved with the segment of data from the chosen channel, to produce a series of CB artefact peaks (Fig. 1, right panel, C). Finally, these peaks are then detected by an automated search procedure for all peaks in the convolved signal with a minimum peak amplitude prominence of two standard deviations above the mean and an inter-peak distance of at least 4/5ths of the length of the mean artefact Fig. 1, right panel, D). This was chosen to allow for small variations in the inter-peak distance without allowing too much leniency that could lead to false peak labelling. These peaks are then taken as the CB artefact onsets for this segment of data. This procedure is then repeated across all segments, producing a vector of detected CB artefact onsets concatenated across segments.

## Method validation

For details concerning the dataset used to assess the validation of our methods, see Additional Information. Artefacts in the EEG were detected by canonical methods as well as by our methods, and their performance at artefact detection compared. Performance of GS artefact detection methods was measured based on sensitivity and specificity. Performance of CB artefact detection methods was measured based on sensitivity, specificity, kurtosis and physiological accuracy. For details of how these measures were calculated, see Additional Information.

### GS artefact detection: performance of canonical approach vs. our approach

The canonical approach to GS artefact detection is to directly record the MR volume/slice onsets at the time of data collection. As the onsets sent from the MR scanner to the EEG recording system indicate the timepoint at which the gradient switches occurred for a new volume/slice acquisition, these onsets necessarily have a 100% sensitivity and 100% specificity, as markers of the true artefact onsets from the origin of the artefacts themselves.

Across all subjects, our alternative approach showed a 100% sensitivity and 100% specificity. As our approach labels the peaks of the GS artefacts directly in the EEG data and doesn't rely on onsets sent from the MR scanner, any transient shifts in the exact sampling point (as can occur when the MR and EEG clocks become desynchronised) at which the artefact onset is logged in the EEG data has no effect on our method. From these results we can conclude that our method is a good alternative for data collected without any pre-recorded GS onsets from the MR scanner at the time of data acquisition.

### CB artefact detection: performance of canonical approach vs. our approach

Descriptive statistics for all performance measures for both the canonical approach and our approach can be seen in Table 1 under Additional Information. The sensitivity and specificity percentages for both approaches were non-normally distributed and hence the Wilcoxon signed rank tests were applied. Results showed a significant difference between the canonical approach and our approach in the sensitivity of the estimated CB artefact onsets (*z*=−2.937, *p* = 0.0033). Median values show an increased sensitivity for our approach (99.54%) as compared with the canonical approach (98.72%). In terms of specificity, there was a non-significant difference (*z* = 1.6636, *p* = 0.0962) between the canonical approach (median = 100%) and our approach (median = 99.11%).

**Table 1:** Performance of cardioballistic artefact detection: Canonical versus our suggested approach. Performance indices of artefact onset estimation for the canonical approach versus our approach are presented across subjects: Sensitivity (%), specificity (%), kurtosis of the first derivative of estimated artefact onsets relative to the expected value under normal conditions (i.e., the ‘baselined kurtosis value’) and physiological accuracy of the first derivative of estimated artefact onsets (%). Mean, the standard deviation (STD) and median across subjects are also shown.

	Our Method				Canonical Method			
Subject	Sensitivity	Specificity	Kurtosis	Physio. Accuracy	Sensitivity	Specificity	Kurtosis	Physio. Accuracy
1	100.00	99.23	33.96	76.45	98.84	100.00	−1.12	76.56
2	100.00	100.00	1.21	100.00	96.60	97.51	8.52	98.44
3	99.67	99.34	37.29	99.67	97.36	98.33	−0.36	100.00
4	99.43	98.87	17.15	99.44	97.45	99.42	0.01	100.00
5	99.74	99.21	45.69	99.21	97.35	100.00	33.97	97.82
6	99.71	98.55	31.25	98.84	99.12	100.00	−0.50	100.00
7	95.77	96.79	−1.94	2.14	96.79	97.31	−1.03	2.15
8	99.70	99.10	25.00	99.40	98.80	100.00	−1.13	100.00
9	99.02	98.05	11.12	95.13	95.03	95.35	8.43	93.69
10	98.92	98.12	8.45	98.66	91.58	92.58	2.01	99.73
11	99.42	99.14	11.18	99.42	99.42	100.00	−1.03	100.00
12	99.02	98.37	0.43	97.39	99.02	100.00	2.98	97.68
13	99.58	99.58	−1.28	31.09	99.16	100.00	−1.23	31.36
14	99.79	98.94	39.94	91.08	98.07	100.00	295.02	99.78
15	99.11	98.67	9.89	1.33	99.11	99.55	36.90	1.35
16	99.38	99.07	27.48	99.69	99.38	100.00	2.91	100.00
17	99.50	99.25	19.09	98.51	98.75	100.00	4.98	100.00
18	99.62	98.87	23.74	80.83	99.62	100.00	2.71	84.35
19	99.41	99.11	15.49	99.70	99.11	100.00	−0.79	100.00
20	93.56	95.00	0.44	57.31	97.39	100.00	−0.67	61.69
21	100.00	99.49	31.13	99.49	99.74	100.00	−1.07	100.00
22	99.34	99.34	7.92	98.34	96.99	97.97	41.09	97.64
23	98.14	97.83	5.87	98.76	98.45	99.69	4.52	98.43
24	99.74	99.74	1.41	99.48	98.68	100.00	−1.49	100.00
25	100.00	99.67	22.89	99.33	98.66	100.00	−1.59	99.66
26	100.00	100.00	−1.10	100.00	99.04	99.68	3.72	100.00
**MEAN**	**99.14**	**98.82**	**16.30**	**85.41**	**98.06**	**99.13**	**16.76**	**86.17**
**STD**	**1.42**	**1.05**	**14.37**	**29.25**	**1.74**	**1.77**	**58.03**	**29.23**
**MEDIAN**	**99.54**	**99.11**	**13.34**	**98.80**	**98.72**	**100.00**	**1.01**	**99.69**

**Fig. 2:**
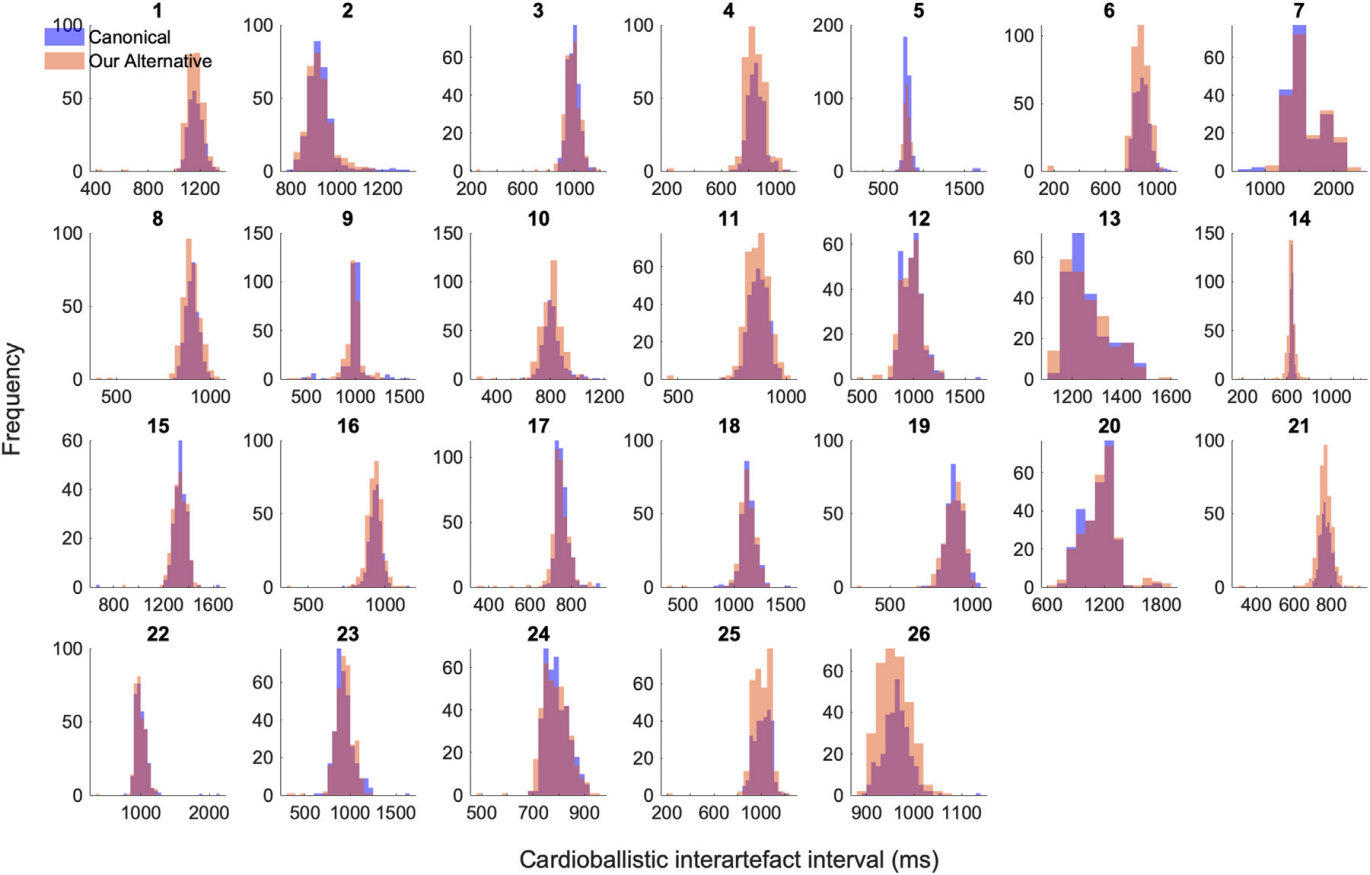
Distributions of estimated cardioballistic inter-artefact intervals: Canonical method versus our method. Per subject, a histogram of the first derivative of estimated cardioballistic artefact onsets is presented. In blue are those estimated by the canonical approach; in orange are those estimated by our approach. Across subjects there is a good overlap between the two approaches, showing a similar performance.

The reference kurtosis value of inter-beat intervals across outside-scanner ECG recordings as a mean across subjects was 4.0833. This value suggests a distribution of inter-beat intervals across subjects that is slightly sharper than a normal distribution. For both approaches, the baselined kurtosis values of inter-artefact intervals across subjects were found to be non-normally distributed and hence a Wilcoxon signed rank test was performed. Bearing in mind the multiple-tests corrected p-value of 0.0125, there was no significant difference between the two approaches in terms of the baselined kurtosis values of inter-artefact interval distributions across subjects (*z*=−2.0445, *p* = 0.0409). Distributions of inter-artefact intervals of the estimated onsets for both approaches for each subject are shown in Fig. 2 under Additional Information.

The physiological accuracy of estimated CB onsets (i.e., the median percentage of inter-artefact intervals that fell inside the expected physiological range) for the canonical approach was 99.69% and 98.80% for our approach. Accuracy percentages across subjects were non-normally distributed for both approaches and the Wilcoxon signed rank test showed no significant difference between the two approaches in terms of physiological accuracy of estimated inter-artefact intervals using the corrected *p-*value for multiple tests (*z* = 2.0588, *p* = 0.0395).

Two of the subjects showed extremely low physiological accuracy across both canonical and our method of CB artefact detection; inspection of the ECG signal recording in the standalone EEG data outside of the scanner showed that this result was because the inter-beat interval range for these subjects was outside of the expected range for young healthy subjects (600 ms to 1200 ms). Subject 7 showed an inter-beat interval ranging from 1190 ms to 1590 ms, and subject 15 showed a range from 1200 ms to 1400 ms.

### Advantages of our methods and tips

Our method holds certain advantages beyond the canonical approach for CB artefact estimation. Most importantly, as our method doesn't rely upon an ECG recording, the long ECG electrode cord that can be potentially dangerous in the MR setting can be removed entirely from the acquisition protocol. Our method does not make any assumptions as to the delay time between the cardiac cycle onset and the onset of the artefact in the EEG. Canonical approaches of artefact detection as implemented in the EEGLAB toolbox assume a fixed delay time of 0.21 s, but intra- and inter-subject variability in this delay time has been previously shown [12]. Fixing this value can only reduce the performance of subsequent CB artefact removal procedures, as a jitter in the exact point of the artefact cycle that is labelled across the onsets will exist. Our approach requires no such assumption, as it labels the artefact onsets directly from the EEG. Using the onsets as detected via our approach, the user can go on to apply canonical artefact removal algorithms as normal.

A further advantage of our approach, given the fact that the CB artefact is highly variable across time and space, is the fact that it allows a flexible segmentation of the data (flexible in terms of segment length) and takes advantage of all recorded channels to find the channels across time that best represent the CB artefact. The length of the segment chosen will depend on the quality of the acquired data. In the case of high-quality data, where the CB artefacts are strongly present consistently across time, then a long segment length can be selected (e.g., 1 min or above). In the case of low-quality data, e.g., with subject motion present, where the artefact is highly variable in shape and amplitude across time and space, the segment length can be set quite short (e.g., 20 s or less). The shorter the segment, the better the detection, but the more manual work required as each segment requires a manual labelling of two artefact peaks) and hence the longer the detection procedure takes. Our method can also be applied to single channel data, but of course then at least two occurrences of the artefact must be visibly detectable in this channel across all segments, i.e., the channel recording should be at a frontotemporal site where the artefact can be reliably seen [12]. Frontotemporal channels are the recommended electrode sites for detection of the CB artefacts.

## Conclusion

In conclusion, we have presented some alternative approaches to the detection of gradient switch and cardioballistic artefacts in EEG data acquired simultaneously with MRI in the absence of additional signals (gradient switch onsets recorded at the time of data acquisition and an ECG signal) that are critical for canonical approaches. Our approaches perform comparably well to canonical approaches and come with specific advantages.

Our proposed methods are useful for EEG datasets acquired in the MR environment without simultaneous ECG or carbon wire loop recordings, where many of the EEG recordings saturated due to inadequate amplifier settings during data acquisition, leaving as little as one useable channel for artefact detection. Optimally, however, one would directly measure the artefacts induced in the EEG data synchronously with data acquisition. A highly promising approach is the addition of carbon wire loops to EEG caps that are electrically insulated from the scalp of the subject, which are constructed to serve the purpose of measuring various artefacts (motion, helium pump or CB) at the time of data acquisition [1] and shown to improve artefact correction as compared with post-processing techniques [33]. Such measurements would be highly advantageous and would drastically improve one's ability to remove the many artefacts that are unfortunately fraught in EEG acquired simultaneously with MRI, including the cardioballistic artefact that post-processing correction techniques cannot fully remove due to its spatiotemporal variability.

## Additional information

### Description of data used for method validation

Data previously acquired from 31 healthy subjects (mean age 27 years, 17 female) were used to assess the validity of our proposed methods. All subjects had no history of neurological/psychiatric disorders and were not taking any psychotropic medication. Conventional clinical MRI assessment (FLAIR, T1, T2) confirmed the absence of any structural brain abnormalities. Two subjects were excluded due to missing MR gradient onset triggers in the recording. Another subject was excluded due to unsuccessful synchronisation between the EEG and MR clocks during data acquisition, and a further two subjects were excluded due to ongoing and extensive motion artefact throughout the recording. In conclusion, 26 subjects’ datasets were used for method validation.

### EEG data acquisition: simultaneously with fMRI

EEG was recorded for five minutes using a 64-channel MR-safe 10–10 system, including an ECG channel (BrainAmp MR plus, Brain Products, Munich, Germany). Data were acquired with a sampling rate of 5000 Hz, an online low-pass filter of 250 Hz, an online high-pass filter of 0.01 Hz and the reference/ground channels were located at FCz and AFz, respectively. Initial impedances were set below 10kΩ and maintained below 50kΩ throughout the recording. Amplifier settings were as follows: range ±16.384 mV, resolution 0.5μV/bit.

### EEG data acquisition: non-simultaneously as a standalone measure

Sixty seconds of eyes-closed data were also recorded outside of the scanner, in the supine position, directly before the simultaneous EEG-fMRI data were acquired. The same EEG equipment and settings as detailed in 3.2.1. were used. The ECG recordings from this data were utilised for the calculation of a reference measure of heart rate in one of the performance indices (physiological accuracy) of CB artefact detection.

### fMRI data acquisition

Simultaneous EEG-fMRI data were acquired in a 3T Philips Ingenia MR scanner (Philips Healthcare, Best, The Netherlands) for five minutes using a 32-channel head coil. Functional, clinical diagnostic and anatomical images were acquired from each subject. Although this study only utilises the EEG data, details of the functional imaging acquisition are provided as they pertain to the nature of the artefacts being detected in this study. Functional data consisted of 150 vol, acquired using a single-shot echo-planar imaging sequence (EPI factor = 31; SENSE factor = 2; TR = 2000 ms; TE = 30 ms; flip angle = 70°; number of slices = 36 with a 0.2 mm slice gap and transverse orientation; voxel size = 3 mm3, field-of-view = 192 × 192 × 115 mm; slice thickness = 3 mm; matrix size = 64 × 62). Subjects were instructed to simply close their eyes and keep still during the 5-minute resting-state period.

### Method validation: sensitivity and specificity of artefact onsets

All analyses were conducted in MATLAB v.2021 (MathWorks) and utilised both custom-made scripts and the fMRIB toolbox in EEGLAB [7,25]. The sensitivity and specificity of the detected GS/CB artefact onsets were calculated and compared between the canonical and our approaches. Detected GS/CB artefact onsets as detected from both canonical and our approaches were manually inspected for false positives and false negatives. Since it is the consistency in the labelled point of each artefact that is important for subsequent artefact removal, the ‘correct’ location of each onset was based on the location of the first onset with respect to the artefact cycle. For example, in the case of the GS artefact, the onsets from the canonical approach (i.e., MR trigger onsets) would be expected to consistently label the artefact just after the arrival of the artefact peak in the EEG, due to a set delay between the beginning of slice/volume acquisition and the transmission of the onset from the MR scanner to the EEG system. The MR trigger onsets necessarily have a sensitivity/specificity of 100%, as the ground truth of artefact onset. Using our approach, the onsets would be expected to consistently label the peak of the GS artefact directly in the EEG. Sensitivity was calculated as the number of true positives / (true positives + false negatives) and specificity was calculated as the number of true positives / (true positives + false positives), following previous methods [12,25]. For sensitivity and specificity values following a normal distribution across both approaches, a paired *t*-test was used to assess the significance of the difference in performance between the canonical and our approach across subjects, for both GS and CB artefact onset detection. Data normality tests across all analyses was performed using the one-sample Kolmogorov-Smirnov test. In the case of data non-normality, a Wilcoxon signed rank test was used. As multiple tests were performed, a Bonferroni correction was applied to results (adjusted *p* threshold of 0.0125).

### Method validation: kurtosis of CB inter-artefact interval

The kurtosis of the distribution of the first derivative of the detected CB artefact onsets was calculated and compared between canonical and our approaches. Kurtosis is indicative of ‘outlier-proneness’, i.e., the weight of the tails of the change in CB artefact onsets’ distribution relative to the normal distribution. A normal distribution has a kurtosis of 3. Longer/heavier tails (more extensive outliers) than those from the normal distribution would lead to a kurtosis value greater than 3, whereas a sharper distribution with lighter tails/less outliers would relate to a kurtosis value less than 3.

The optimal control value for kurtosis of CB inter-artefact interval was derived from the ECG measurements outside the scanner. We attained a mean kurtosis value of inter-beat intervals across subjects from the 2-minute outside-scanner ECG recordings. The data was high-pass filtered to above 1 Hz (FIR, transition bandwidth 1, order of 5) and the R-peaks of the QRS complex could be easily detected using an automated peak detection method using the ‘findpeak’ algorithm as provided in MATLAB. Two thresholds were applied for the peak detection across all subjects but one: a minimum peak threshold of 150uV and a minimum peak distance of 300 ms. One subject required a minimum peak threshold of 400uV for accurate R-peak detection. Peaks were visually checked. A mean kurtosis value across subjects was taken as the baseline kurtosis value as a reference for optimal CB artefact onset detection performance.

The difference between the kurtosis value of the CB artefact onsets and this reference kurtosis value was taken as the outcome measure of artefact detection performance, for both the canonical approach and our approach to CB artefact detection, referred to as the ‘baselined kurtosis value’. This gave us baselined kurtosis values, per subject for each approach, that indicate to what extent the shape of the distribution of estimated inter-artefact intervals differs from the mean distribution shape that can be expected as a baseline across subjects. Optimal CB artefact onset detection would be indicated by a baselined kurtosis value close to 0. A baselined kurtosis value greater than 0 would indicate a sharper distribution than expected from the baseline outside-scanner ECG recordings, potentially indicating insufficient representation of inter-beat variability. A value less than 0 would indicate a heavier-tailed distribution more prone to outliers than expected, indicative of the effect of false positives/negatives on the distribution.

The significance of the difference in performance of our approach versus the canonical approach in CB artefact detection in terms of the baselined kurtosis values was analysed across subjects. If distributions were normally distributed, a paired *t*-test was used, otherwise a Wilcoxon signed rank test was applied.

### Method validation: physiological accuracy of CB inter-artefact interval

A final indicator of performance of the CB artefact detection is the number of CB artefact onsets with inter-artefact delays outside the expected physiological range. The expected physiological range was 600 ms to 1200 ms for healthy subjects [12]. All CB artefact onsets with an inter-artefact interval outside the expected range were labelled as inaccurate, and the percentage of accurate onsets between the canonical and our approaches were compared for a significant difference using a Wilcoxon signed rank test.

## Ethics statements

Subjects gave informed consent, and the acquisition of the data was ethically approved by the local ethics committee of Technical University of Munich, Germany and conformed with relevant guidelines and regulations.

## CRediT authorship contribution statement

**Rachel Nuttall:** Conceptualization, Methodology, Formal analysis, Validation, Visualization, Writing – original draft, Writing – review & editing. **Aya El Mir:** Software, Writing – review & editing. **Cilia Jäger:** Investigation, Data curation, Writing – review & editing. **Svenja Letz:** Formal analysis, Validation, Writing – review & editing. **Afra Wohlschläger:** Data curation, Methodology, Writing – review & editing. **Gerhard Schneider:** Conceptualization, Supervision, Project administration, Writing – review & editing.

## Declaration of Competing Interest

The authors declare that they have no known competing financial interests or personal relationships that could have appeared to influence the work reported in this paper.

## Data Availability

Data will be made available on request.
